# Accessibility and Acceptability of Infectious Disease Interventions Among Migrants in the EU/EEA: A CERQual Systematic Review

**DOI:** 10.3390/ijerph15112329

**Published:** 2018-10-23

**Authors:** Matt Driedger, Alain Mayhew, Vivian Welch, Eric Agbata, Doug Gruner, Christina Greenaway, Teymur Noori, Monica Sandu, Thierry Sangou, Christine Mathew, Harneel Kaur, Manish Pareek, Kevin Pottie

**Affiliations:** 1 Bruyère Research Institute, 85 Primrose Ave, Annex E, Ottawa, ON K1R 6M1, Canada; matt.driedger@uottawa.ca (M.D.); amayhew@bruyere.org (A.M.); sandumonique@yahoo.com (M.S.); thiesang@gmail.com (T.S.); cmath054@uottawa.ca (C.M.); hkaur006@uottawa.ca (H.K.); 2 Departments of Family Medicine & Epidemiology and Community Medicine, University of Ottawa, Ottawa, ON K1H 8M5, Canada; vivian.welch@uottawa.ca (V.W.); gruner18@yahoo.ca (D.G.); 3 Department of Paediatrics, Obstetrics, Gynaecology and Preventive Medicine, Universität Autònoma de Barcelona, 08193 Barcelona, Spain; ericagbata@gmail.com; 4 Division of Infectious Diseases, Jewish General Hospital, McGill University, Montreal, QC H3T 1E2, Canada; ca.greenaway@mcgill.ca; 5 Centre for Clinical Epidemiology of the Lady Davis Institute for Medical Research, Jewish General Hospital, Montreal, QC H3T 1E2, Canada; 6 European Centre for Disease Prevention and Control, 16973 Stockholm, Sweden; teymur.noori@ecdc.europa.eu; 7 Department of Infection, Immunity and Inflammation, University of Leicester, Leicester LE1 7RH, UK; mp426@le.ac.uk

**Keywords:** access to care, disease prevention, public health, stigma, refugees, migrants

## Abstract

In the EU/EEA, subgroups of international migrants have an increased prevalence of certain infectious diseases. The objective of this study was to examine migrants’ acceptability, value placed on outcomes, and accessibility of infectious disease interventions. We conducted a systematic review of qualitative reviews adhering to the PRISMA reporting guidelines. We searched MEDLINE, EMBASE, CINAHL, DARE, and CDSR, and assessed review quality using AMSTAR. We conducted a framework analysis based on the Health Beliefs Model, which was used to organize our preliminary findings with respect to the beliefs that underlie preventive health behavior, including knowledge of risk factors, perceived susceptibility, severity and barriers, and cues to action. We assessed confidence in findings using an adapted GRADE CERQual tool. We included 11 qualitative systematic reviews from 2111 articles. In these studies, migrants report several facilitators to public health interventions. Acceptability depended on migrants’ relationship with healthcare practitioners, knowledge of the disease, and degree of disease-related stigma. Facilitators to public health interventions relevant for migrant populations may provide clues for implementation. Trust, cultural sensitivity, and communication skills also have implications for linkage to care and public health practitioner education. Recommendations from practitioners continue to play a key role in the acceptance of infectious disease interventions.

## 1. Introduction

Migrant populations often come from or travel through low- and middle-income countries where the prevalence and burden of infectious diseases differs from the European Union/European Economic Area (EU/EEA) [1]. Migrant populations include immigrants, refugees, asylum-seekers, displaced persons, undocumented migrants, and other foreign-born residents. In the EU/EEA, for example, subgroups of migrants have a higher prevalence of HIV, tuberculosis (TB), hepatitis B (HBV), and hepatitis C (HCV), and have lower rates of childhood vaccinations compared to native-born populations [1].

Evidence-based guidelines can direct public health and healthcare practitioners in the screening and treatment of such diseases. These guidelines include information on testing and vaccination and may also consider culturally sensitive ways to approach migrants. For example, existing guidelines for HIV among migrant populations [2,3,4] synthesize evidence on benefits, harms and cost effectiveness, and also provide some interpretation on qualitative data relevant to HIV related stigma and strategies to link patients for treatment. To implement public health guidelines, an understanding of migrant populations’ perceptions and fears is needed [5]. Thus, to ethically offer interventions, we need to understand the perspective of migrants regarding the acceptability of interventions, value placed on outcomes, and accessibility of screening and treatment of infectious disease interventions in the EU/EEA [6,7].

The acceptability of infectious disease interventions influences the readiness of migrants and clinicians to incorporate guidelines into practice, as seen in the case of HIV screening [8]. Insufficient knowledge among clinicians about the acceptability of interventions may inhibit them from offering screening to migrants [9]. How patients value the disease-related outcomes of interventions (e.g., perception of risk of disease, diagnoses, symptoms, or disease resolution), or other outcomes (e.g., time away from work, stigma, side effects, or adverse events) can create barriers to the uptake of guideline recommendations [5]. For example, one qualitative study on developing decision aids for HIV testing for newly arrived Sub-Saharan African women to Canada demonstrated how the provision of accurate HIV information can reduce stress [10]. Existing strategies to improve access to healthcare for migrants include support for transportation, interpreters, and cultural brokers [11]. 

The objective of this study is to understand the acceptability, the value placed on outcomes and the accessibility of infectious disease interventions and other health services among recently arrived EU/EEA migrants. We focused specifically on tuberculosis, HIV, HBV, HCV, vaccine-preventable diseases (VPD), and parasitic diseases; diseases that were selected during an ECDC consensus meeting in Stockholm [12]. We also aimed to explore how the GRADE CERQual tool can appraise qualitative research on implementation considerations. 

## 2. Materials and Methods 

### 2.1. Search Strategy and Selection Criteria

We conducted a systematic review of qualitative reviews, and adhered to the Preferred Reporting Items for Systematic Reviews and Meta-Analyses (PRISMA) reporting guideline [13]. A team of experts with qualitative research expertise developed a protocol that considered implementation for public health interventions relevant to migrant populations in EU/EEA. We registered the protocol on Prospero (CRD42016045798) and published our detailed review methods in BMJ Open [12,14]. 

We searched MEDLINE, MEDLINE In-Process, MEDLINE Ahead of Print, EMBASE, CINAHL, DARE, and CDSR for articles published between 1 January 2010 and 29 July 2016. The full search strategy is provided in Supplementary File S1. We also searched grey literature for published reports that met our inclusion criteria from the CDC, ECDC, UNAIDS, EU, and WHO, and scanned references to identify additional qualitative systematic reviews. We included qualitative systematic reviews that reported on values, perceptions on access, and acceptability of infectious disease interventions (see Appendix A). We restricted our inclusion to studies published in English. We included reviews if search and selection strategy methods were explicitly provided, and if the review included qualitative evidence. We focused on migrant and forcibly displaced populations, including children, adolescents, pregnant women, and adults. See Appendix B for full inclusion and exclusion criteria. 

### 2.2. Study Selection and Data Extraction

Three independent team members (MD, MS, TS) screened title and abstracts in duplicate, followed by full-text assessments for eligibility. Conflicts were resolved through discussion or the involvement of a fourth reviewer (AM). Data were downloaded into EndNote reference software [15]. We assessed the methodological quality of included reviews using the Assessing Methodological Quality of Systematic Reviews tool (AMSTAR) [16] but did not exclude any studies based on quality. 

The same team members extracted data from the included reviews in duplicate. We used a calibration exercise prior to data extraction and discrepancies were resolved through discussion. We designed our data extraction form using the Jacob’s accessibility framework [17]. The Jacob’s accessibility framework highlights barriers to accessing health services from both the supply and demand side, and as such recognizes that determinants of geographic accessibility, acceptability, availability, and affordability play a critical role in access. The framework focusses more on accessibility rather than appraising the acceptability and attitudes towards these services. However, adapting this framework to create an inclusive data extraction form (see Appendix C) allowed us to capture all relevant data, which was subsequently contextualized with respect to our research objectives. 

### 2.3. Data Synthesis

We contextualized the preliminary findings on migrant populations using the Health Belief Model framework (HBM) [18]. The HBM is a commonly used model of the beliefs, expectations, and values that underlie preventive health behavior [19], and was therefore selected for its clear alignment with our stated research objectives involving the values and acceptability of interventions. HBM suggests that six factors predict health behavior: perceived susceptibility, perceived severity, benefits to action, barriers to action, self-efficacy, and cues to action [18]. 

We applied a qualitative lens considering saturation (200 studies were identified within the reviews) and triangulation of data between different diseases, migrant populations, and destination countries to identify preliminary findings. We consulted clinicians (KP, MP, DG, CG) with expertise and experience in migrant health to identify and corroborate the credibility, transferability, confirmability, and dependability to establish the trustworthiness of these findings. Of note, while many reviews discussed how knowledge of risk factors influences health behavior, only two reviews [20,21] commented specifically on how susceptibility, in itself, determines health behavior, which is how “perceived susceptibility” is classically theorized in the HBM [18]. Given the strong cognitive component of susceptibility within the HBM [22], we opted to include the knowledge data in our main findings, yet we typified this as “knowledge of risk factors” to maintain accuracy.

Five of the 12 preliminary findings were selected as “key findings” to be further analyzed with the Confidence in the Evidence from Reviews of Qualitative research (CERQual) tool. These were selected by consensus among three authors (MD, KP, AM), based on their respective strength of evidence, the number of reviews supporting the finding, the level of variability in review findings, and the significance of the findings as stated in the included reviews.

We used the CERQual tool to assess the confidence of our findings. CERQual is a new method for assessing the confidence of qualitative review evidence, similar to how the GRADE approach assesses the certainty of quantitative evidence [23]. CERQual bases this evaluation on four criteria: (a) methodological limitations of included studies supporting a review finding, (b) the relevance of included studies to the review question, (c) the coherence of the review finding, and (d) the adequacy of the data contributing to a review finding. To our knowledge, CERQual has not been used in a review of reviews to date. To apply the principles of CERQual to a review of reviews, we needed to make minor adjustments, such as considering the number of primary studies within a given review to assess the adequacy criterion. 

## 3. Results

### 3.1. Study Selection

**Figure 1:**
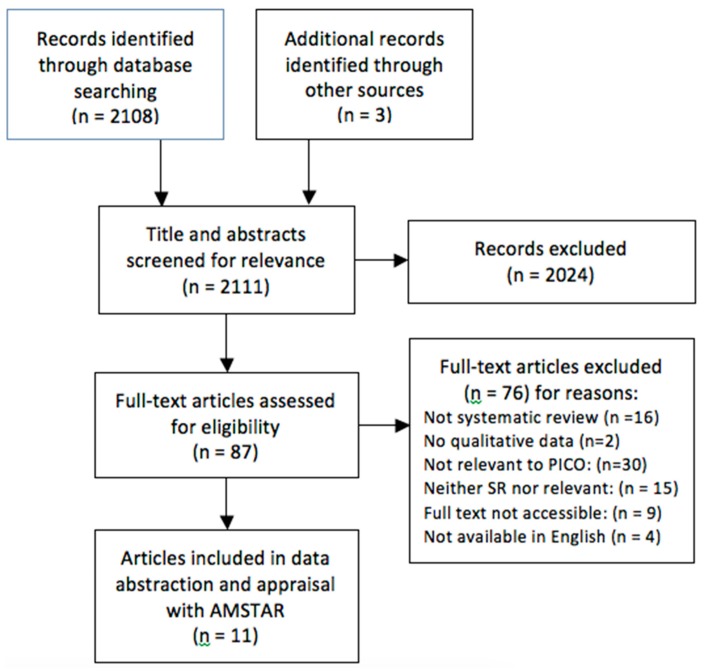
PRISMA Flow Diagram.

The formal search identified 2108 articles. Reference scanning identified three additional reviews. We screened 87 full-text articles and 11 qualitative systematic reviews met our inclusion criteria. All reviews examined populations migrating from low- and middle-income countries to high-income countries. See PRISMA Flow Sheet showing selection, Figure 1.

Three of the systematic reviews focused exclusively on migrant populations [24,25,26]. Other reviews examined migrant populations as subgroups within the general populations [21,27,28]. The host population countries were predominantly in Western Europe and the United States. Participants mostly consisted of Latino, Hispanic, or sub-Saharan African migrants, but also included South-East Asian and Middle-Eastern migrants. Most reviews included both quantitative data from cohort and cross-sectional studies as well as qualitative data from focus groups and interviews. Three reviews focused on HIV, three on HBV/HCV, and five on TB. No reviews specifically addressed vaccine-preventable or parasitic diseases. See characteristics of included studies in Table 1. 

### 3.2. Methodological Quality

We assessed methodological quality using the AMSTAR tool. AMSTAR was originally designed for quantitative reviews but many of the criteria are applicable to qualitative reviews, such as, a priori design, duplicate selection, comprehensive search, criteria, and characteristics of included and excluded studies and consideration of scientific quality. The authors have used AMSTAR for qualitative systematic reviews [33,34]. AMSTAR scores were distributed fairly evenly between one and seven points out of a possible 11, with a median score of 2/11. AMSTAR items varied significantly with respect to the proportion of reviews meeting that item. 

### 3.3. Migrants’ Perceptions of Acceptability

We organized the findings using the Health Beliefs Model (HBM) [19]. Through our framework analysis, we identified 12 preliminary findings from the data. See Table 2 for a detailed description of these findings.

Three reviews reported on acceptability of interventions [25,26,28]. Tomas et al. found that the TB screening process was generally well-received among migrants [26]. According to Mitchell et al. [28], the overall acceptability of TB screening among migrants was considered to be high, yet migrants’ perception of TB as a severe disease was associated with screening refusal. Owiti et al., reported that some migrants expressed motivation to or actively sought screening for HBV/HCV, and that certain populations were receptive to HBV vaccination [25]. 

Furthermore, peer support and the influence of family members promotes self-efficacy in seeking healthcare and improves the acceptability of interventions, yet there are also instances in which these social connections may introduce other barriers [20,24,25]. For example, family support would improve adherence to TB treatment, but the need for women, at times, to request their partner’s approval to seek screening acted as a barrier [20,24]. Cultural and family beliefs that differ from those of the host nation may present a perceived barrier, and may lead to other barriers, such as disease-related stigma, that can influence acceptability of care [21,26,27,30,31,32]. In addition, various attitudes towards an intervention itself, especially side effects and cultural taboos, may influence its acceptability among migrants [24,26,27].

The patient-practitioner relationship was consistently emphasized as an important cue to action in seeking further care. Trust, cultural sensitivity, and communication skills can greatly improve the acceptability of infectious disease interventions [20,25,26,27,28,29,30,31]. Therefore, recommendations from healthcare practitioners can influence migrants’ health seeking behavior [25,30,31].

Social determinants also influenced the acceptability of interventions. The number of years of formal education was positively correlated with HIV screening [21,24], HBV/HCV knowledge, [25] testing and vaccination [30] and TB screening and treatment [27]. In one review, older age was associated with HBV/HCV knowledge [25], but another review, among Asian Americans/Pacific Islanders [30] showed younger age was associated with HBV/HCV knowledge. Gender also played a role, as females were more receptive to HIV screening [20,24], but males were more likely to be screened for HBV [30,31].

### 3.4. Migrants’ Values on Outcomes of Interventions

Traditional beliefs of migrants may play a role in the value placed on outcomes of infectious disease interventions. The reviews report that migrants’ perceived severity of and susceptibility to infectious diseases influences their uptake of testing and treatment interventions. Reviews of TB, HIV and hepatitis reported a low level of western knowledge and understanding of risk factors and transmission of disease among migrants, and this may make them less likely to seek screening, vaccination, or treatment [20,21,24,25,27,29,30,31]. While the degree of knowledge varied among studies, it was consistently associated with the uptake of interventions. 

Migrants reported certain perceived benefits as valued outcomes of screening, vaccination, and treatment. The most consistently valued outcomes included reassurance of disease-free status and thus prevention of transmission to others [21,24,26,30]. Uptake of interventions was associated with perceptions of negative disease-related outcomes among migrants. Stigma, and its related connotation, acts as a large barrier to screening and treatment [20,21,24,25,26,27,29,32]. Indirect costs, such as loss of employment and loss of migration status and social status, reduced the value placed on interventions [20,24,25,29,32]. For example, certain migrants feared that a positive test result would have a negative impact on their immigration status or refugee claim. Symptoms were consistently reported as an important cue for health actions; for example, migrants value screening or treatment of symptomatic diseases over asymptomatic diseases and often wait until they are symptomatic before seeking care [24,26,29,30,31]. 

### 3.5. Accessibility of Health Services

Barriers to accessibility were reported at both structural and community levels. Structural barriers to care for migrants include cultural and language barriers [35], inadequate practitioner cultural competencies [36], disease-related stigma and discrimination [20], perceptions of health and healthcare [37], and legal status of migrants [24]. Community-level barriers include the availability and awareness of services such as transportation, economic barriers including healthcare coverage and cost of services, and policy barriers such as the healthcare system capacity and coverage. These barriers interact with poverty, inequality, and power, further exacerbating the poor health of the migrants [38]. Time spent accessing healthcare can incur a significant opportunity cost for migrants, especially when they have insecure employment or cannot meet basic needs during their settlement process [20,24,26,27,28,29]. Furthermore, barriers related to the migration process, including language proficiency, cultural barriers, and navigation of the healthcare system, can make interventions less acceptable or accessible for migrants [20,21,24,25,26,27,29,30]. While interpreters may improve accessibility, their presence may introduce new potential barriers surrounding confidentiality [24,26,30]. 

### 3.6. Confidence in Findings 

We analyzed the confidence of our five findings using CERQual (see Table 3). Three findings were assigned a moderate confidence rating, and two were assigned a low confidence rating. See Table 4 for a detailed explanation of confidence ratings.

## 4. Discussion

We identified 11 systematic reviews that addressed factors influencing acceptability, the value placed on outcomes, and accessibility of screening and treatment of infectious diseases among migrants. Using the framework of the Health Belief Model, we found factors that influenced healthcare engagement and intervention uptake in each disease group, i.e., TB, HIV, HBV, and HCV. This analysis supports the role of the HBM in identifying and organizing implementation considerations in public health guidelines for migrants. We also assessed the confidence in five key findings using the CERQual tool. Three findings were rated as moderate confidence, and two were rated as low confidence (See GRADE CERQual Table 4). 

The findings of this review suggest that disease-related stigma, and inaccurate knowledge related to certain infectious diseases, continue to be major deterrents for screening among migrants. However, ongoing education of migrant patients and their physicians may increase adherence to TB screening and treatment [27]. Stigma relates to traditional and western beliefs concerning disease outcomes, and these beliefs interact with longstanding cultural and social barriers [37]. Stigma can manifest in family and community life and may impact employment as well as healthcare. Addressing stigma will require a multi-faceted approach that involves engagement of affected communities as well as efforts to reduce structural barriers [24], as exemplified by the integration efforts taking place in Germany [39]. 

Migrant populations face screening at the political, public health and primary health care levels. We found that migrants consider the indirect costs that potentially accompany disease results, such as loss of employment and loss of migration status and social status. These negative outcomes may vary across the EU/EEA. On the contrary, migrants value screening, post hoc, when they do not have a disease.

Migrants consistently identify trust in practitioners as a key determinant to accepting infectious disease interventions [40]. Various organizations have developed cultural competency [41], cultural humility [42] programs to build trust for newly arrived migrants. In the context of cultural sensitivity, practitioners’ approach may play an important role for linkage to care for migrants. More research, including participatory research, is needed to engage migrants in implementation strategies [43,44]. For example, one qualitative study used interviews with migrant leaders in community health to not only identify barriers to disease screening, but also identify innovative approaches to mitigate barriers by combining screening for all relevant diseases into one standardized check-up, thereby improving accessibility and further reducing disease-related stigma [45]. 

### 4.1. Implications for Practice

The qualitative data from our 11 reviews reports a compelling story of migrant access to care issues and acceptability issues related to stigma, indirect costs, and health system barriers. When migrants experienced disease symptoms or were able to perceive benefits from screening and/or trusted their practitioners, they were more likely to value, accept, and access infectious disease interventions. These findings tap into the lived experience of many migrants and may have relevance for screening programs; however, these findings cannot be generalized across all populations and diseases.

### 4.2. Strengths and Limitations

Traditionally, the GRADE CERQual tool is used to assess confidence in the evidence of synthesized qualitative studies. This paper is the first to adapt the CERQual tool to assess the confidence of systematic review level qualitative evidence. We also directed our findings and applied our confidence ratings as evidence in the ECDC Guidance development process, including values on intervention outcomes and acceptability of screening and treatment interventions of infectious diseases among migrant populations. These findings were implemented into evidence to decision tables and helped to develop ECDC guidance and implementation considerations for migrants. 

According to the AMSTAR scores, the quality of eligible systematic reviews was low, highlighting a need for more rigorous evidence on the acceptability and accessibility of interventions among migrants. Specifically, the methods used to combine findings were generally appropriate, yet only two reviews [24,29] assessed and documented the quality of the primary studies included. While this may impact the validity of our findings, we demonstrated how the CERQual methodology can be used to account for the quality of the included reviews to generate sound assessments of the strength of qualitative evidence. 

Our systematic review of reviews approach allowed us to use data that summarized findings from over 200 primary studies and supported the assessment of adequacy, consistency, and coherence. However, this approach also created some methodological challenges. We were obliged to report the findings without additional interviews and triangulation. Second, while we used the number of reviews and primary studies supporting a finding as evidence for the robustness of a finding, the precise relevance of these findings varied.

We began with six infectious disease interventions, which were consistent with those prioritized by the EU/EEA guidance work. This allowed us to consider consistencies across different individual diseases and provided more data to synthesize into findings. However, examining the data in aggregate may mask differences between these diseases. For example, most of the evidence on stigma comes from reviews on HIV and TB, and thus may not be generalizable to HBV or HCV or diseases not represented in the included reviews. We were unable to find qualitative systematic reviews that addressed vaccine-preventable diseases or intestinal parasites. While some of the evidence is likely relevant to these diseases, we accept that some of the barriers may be different. For example, VPDs are likely more relevant for migrant children and parents/caregivers, for whom the barriers and facilitators differ from adult migrants.

We were able to look at the findings from various migrant population and destination country perspectives. We chose to group the priority infectious diseases together, demonstrating that migrant perspectives varied across these diseases. We were unable to effectively rule out outliers on all the priority conditions and our findings are more aligned with migrant populations than destination countries.

## 5. Conclusions

Our review highlights migrants’ perspectives on screening and treatment of infectious diseases, and as such, provides insight as to why migrants may accept or reject screening and treatment. Addressing disparities in prevalence and treatment rates of diseases between and within migrant populations will require implementation strategies that address migrant and practitioner knowledge, fear, and access barriers to health services. The acceptability, value of main outcomes and accessibility of screening and treatment interventions among migrants is highly dependent on the cultural sensitivity, relationship with healthcare professionals, disease-related stigma, and the degree of knowledge and self-perceived risk of diseases. Migrants may fear negative outcomes of screening including indirect costs related to the employment and immigration status, and they value screening and treatment less when asymptomatic. While our findings demonstrate similarities and differences across several infectious diseases, the available data was not sufficient for a complete analysis of factors that are specific to individual diseases or to migrant sub-populations. This highlights a need for ongoing implementation research involving individual populations and diseases to address this important public health and primary care topic.

## Figures and Tables

**Table 1:** Characteristics of Included Studies.

Citation	Years Searched	Population	Intervention/Service Setting	Analysis/Synthesis Approach	EU/EEA Settings Included?	1’ Study Design	# of 1′ Studies	AMSTAR Score (/11)
Alvarez-del Arco et al. [20]	2005–2009	Migrants and ethnic minorities populations living in high-income countries Migrants were largely from sub-Saharan Africa and Latin America, (1) and other regions.	HIV testing and/or counselling in health and community settings	None specified-Narrative	Yes	Quantitative (25); mixed-methods (2); qualitative (6); literature reviews (4)	37	1
Blondell et al. [24]	1997–2014	Foreign-born: African, particularly Sub-Saharan, and Hispanic/Latino migrants were the most studied populations.	HIV screening, testing	None specified - narrative	Yes	quantitative (n = 21) (descriptive/non-randomized) and qualitative (n = 10).	31	3
de Vries et al. [29]	2010–2017 (OECD countries); or 1990–2017 (EU, EEA, EU candidate countries)	Hard-to-reach populations including homeless, migrants, travelers (including Roma), refugees, others. 7/10 studies were of migrants only. One study included homeless, migrants, and drug users.	TB services of any kind	Thematic and content analysis	Yes	Qualitative: Interviews (6), focus groups (2), both Interviews and Focus groups (3) multi-method participatory research (1)	12	7
Do et al. [30]	2002–2009	Asian Americans and Pacific Islanders (69% foreign-born).	Health education, screening, and vaccination for HBV	None specified - narrative	No	Cross-sectional (13); RCT (1); quasi-experimental (1); Longitudinal (1)	20	1
Greenaway et al. [27]	1950 to 17 December 2008) *	Immigrants (subgroup).	Screening and treatment of latent TB	Summary of findings table (GRADE)	Not specified	SRs (7) and guidelines (2)	9	2
Mitchell et al. [28]	1985–April 2011	30 individual risk groups * Data extracted from two groups only—Internally Displaced Populations (IDPs), and “Migrants/Immigration”	TB screening (CXR, Mantoux TST)	Metasynthesis	Yes	Qualitative and Quantitative literature.	21	2
Nguyen-Truong et al. [31]	1998–2012	Vietnamese Americans—most studies report that majority of sample are immigrants, but most aggregated immigrant and native-born.	Screening (HBV and Colorectal cancer)	None specified	No	Descriptive (15); Interventional (2); Qualitative (3); Chart/medical record review (2); Mixed-method (1)	23	2
Owiti et al. [25]	1970–2014 **	High-risk 1st- or 2nd-gen immigrants from high-prevalence countries or intermediate prevalence countries who migrated to traditionally low prevalence countries.	Knowledge of HBV and/or HCV infections and/or with targeted screening, vaccination, and treatment	Narrative synthesis	Yes	Quantitative surveys (39) and qualitative studies (11); mixed-methods (1)	51	6
Pottie et al. [21]	1995–2008	Immigrants and refugees (subgroup).	HIV Screening and treatment	Summary of findings table (GRADE)	Not specified	SRs (7) and guidelines (2)	8	4
Tankimovich et al. [32]	1998–2012	Homeless and immigrants with TB.	TB detection and treatment (active and latent)	None specified—narrative	Yes	Quantitative (17); Qualitative (5); Intervention studies (10)	22	2
Tomas et al. [26]	1995–2011	Immigrants, and intra-national migrants and including migrants, asylum-seekers, refugees.	Screening and treatment of TB (active and latent)	Meta-ethnography	Yes	In-depth interviews (24); focus groups (12); participant observation (5); case studies (1); Other (6) Many combined qualitative and quantitative methods.	30	3

* Includes primary studies from 1995 onwards; ** Includes primary studies from 1999 onwards.

**Table 2:** Preliminary Findings from Health Belief Model Framework Analysis.

	Main Theme	Reviews Cited (Lead Authors)	Disease-Specific Supporting Examples
Knowledge of Risk Factors	Low level of knowledge of risk factors and transmission of disease may make migrants less likely to seek screening, immunization, or treatment.	(5) de Vries, Owiti, Lee, Nguyen, Blondell	TB: Underestimated risk of acquiring TB due to poor understanding of transmission and false beliefs, e.g. that TB is not present in US. (de Vries) HBV/HCV HBV screening is associated with better knowledge of HBV and specific modes of transmission (Owiti, Lee, Nguyen) HIV: Migrants with greater knowledge of HIV and its risk factors were more likely to be screened (Blondell)
Perceived Susceptibility	Low perceived personal risk of acquiring an infectious disease may make migrants less likely to seek screening	(3) Greenaway, Pottie, Alvarez	Perceived low risk of progressing from latent to active infection is a barrier to screening/treatment of latent TB (Greenaway) HIV Low perceived personal risk is a barrier to screening (Pottie, Alvarez)
Perceived Severity	The severity and consequences (medical, social, economic) of diseases varied between studies, were generally well understood. However, the literature is divided on whether this is a motivating factor, or a perceived barrier to screening (i.e. risk of realizing the negative consequences through screening).	(4) Blondell, Lin, de Vries, Owiti)	Tuberculosis: TB was thought to be important, potentially fatal disease; participants afraid of disease’s severity (Tomas) Varying perception on TB severity included: very serious, lethal disease, a long-lasting but curable disease, fear of dying from incurable disease (de Vries) HBV/HCV: Perceived outcomes of HepB and C: Poor health; discrimination/stigma; loss of income; loss of social status; liver disease (Owiti) On the other hand, belief that HBV infection is transient could lead to it not being taken seriously (Owiti) HIV: Concerns regarding the logistical consequences of living with a positive status, and fear of a future with a positive result, reduced the acceptability of screening among African migrants (Blondell)
Perceived Benefits	Several distinct, tangible benefits to screening, vaccination, and treatment were reported by reviews, especially reassurance of negative status and prevention of spread to others.	(4) Tomas, Do, Pottie Blondell,	Tuberculosis: In some communities, benefits of treating latent TB were well understood, including efficacy of medication, avoidance of stigma, and reducing risk of transmission to others (Tomas) HBV/HCV: Primary motivations for hepatitis B vaccination were protection of future health and avoidance of hepatitis B (Do) HIV: “Just wanted to find out” was a motivator among Latino migrants; “ensure they were healthy and clean” (Blondell) Refugees and refugee claimants might be reluctant to accept screening tests because they fear limited access to antiretroviral treatment and thus do not see a perceived benefit to screening (Pottie)
Perceived Barriers	Stigma is an overarching barrier to screening and treatment that was reflected in most diseases and reviews. Stigma is also related to other perceived barriers (e.g. confidentiality issues with interpreters, hesitancy to report symptoms to family/healthcare providers)	(8) Tomas, Tankimovich, de Vries, Greenaway, Pottie, Owiti, Blondell, Alvarez,	Tuberculosis: Feelings of stigma influenced immigrants’ attitudes towards prevention and diagnosis and could prevent them from sharing relevant information with their doctors. Medical interpreters often posed a problem due to the perceived sensitivity of the information, loss of privacy, and stigmatization (Tomas) HBV/HCV: Shame and stigma of hepatitis may negatively uptake screening; may dissuade migrants from disclosing test results (Owiti) HIV: Stigma, discrimination related to HIV described as most important impediment to HIV testing, treatment (Pottie) Stigma is not significant across all studies, which may be explained by population characteristics or definitions of stigma. The few quantitative studies on stigma failed to show a statistically significant association with testing (Blondell)
	Time spent accessing healthcare can incur a significant opportunity cost on migrants, especially when they are in a precarious employment situation or do not have basic needs met in their settlement process.	(6) Tomas, Greenaway, de Vries, Mitchell, Blondell, Alvarez	Tuberculosis: Missed days at work is a barrier to TB screening and treatment adherence (Greenaway) Reasons for refusing TB screening were predominantly a lack of time (Mitchel) HIV: Provision of rapid testing outside normal working hours may improve uptake by eliminating the opportunity cost of missed work (Blondell, Alvarez)
	Indirect costs that may be unique to migrants can reduce the value placed on these screening and treatment interventions. The most prominent of these was that a positive test result may have a negative impact on the migrant’s immigration status or refugee claim.	(5) Lin, Tankimovich, Blondell, Alvarez de Vries,	Tuberculosis: Undocumented status was consistently correlated with non-adherence to treatment (Lin) Migrants may not seek treatment due to fear of revealing their illegal immigration status (Tankimovich) HIV: Migrants placed their legal status as among their highest priorities, and fears on the implications of testing positive on their visa/residency application or deportation were main barriers in several studies (Alvarez). However, this was not a barrier in all studies (Blondell)
	Factors inherent to the migration process, including language proficiency, cultural barriers, and navigation of the healthcare system, can create barriers for migrants. However, reviews reported conflicting results regarding the influence of acculturation and language proficiency	(9) Tomas, Lin, Do, Owiti, Pottie, Blondell, Greenaway, de Vries, Alvarez,	Tuberculosis Years spent in host country inconsistently associated with treatment completion/outcomes. Two studies found that immigrants with better English proficiency were at increased risk of not completing treatment (Lin) Lack of familiarity with the local language was a barrier to screening (Tomas) HBV/HCV Access to interpreter services increased odds of testing (Do, Owiti) One study reported an associated between lower English proficiency and higher likelihood of being tested for HBV, while another found that not needing an interpreter was associated with getting tested (Owiti) HIV Non-integration of health services was a key barrier to HIV screening Inability to communicate in the host country’s language was a prominent barrier to screening (Pottie) While language services increase uptake, translators may introduce confidentiality concerns (Blondell)
	Various attitudes and expectations of the intervention itself (the procedure or its side effects) may influence its acceptability among migrants	(4) Greenaway, Lin, Blondell, Tomas	Tuberculosis Barriers to TB screening included fear of a painful test (Tomas) and venipuncture (Greenaway) Side effects are inconsistently associated with treatment adherence. Quantitative studies found no significant correlations in multivariate analysis (Lin) HIV Some African migrants felt that too much blood was taken during screening (Blondell)
Cues to Action	Recommendation from healthcare providers can influence healthcare seeking by migrant patients.	(3) Owiti, Do, Nguyen	HBV/HCV Recommendation by healthcare professionals was positively associated with uptake of screening and vaccination (Owiti, Do, Nguyen)
	The importance of the patient-physician relationship was consistently emphasized. Trust, cultural sensitivity, and communication skills can act as facilitators to the acceptability of infectious disease interventions, whereas a negative relationship can serve as a barrier.	(7) Tomas, Greenaway, Mitchel, de Vries, Do, Nguyen, Owiti	Tuberculosis Using a dedicated nurse and cultural interpreter to provide a “transcultural” approach increased screening acceptability within one year (Mitchell) Health staff can improve adherence to treatment by providing personal advice with sensitivity and “the ability to establish a personal relation on the same cultural terms”. Positive relationships with health staff are perceived as “a crucial element” (Tomas) HCV/HBV Poor patient-doctor communication, and reliance on professional opinion, discouraged testing and vaccine uptake (Do, Nguyen)
	The presence of symptoms can be a necessary cue to seeking healthcare among migrants who may not understand or value the importance of treating asymptomatic disease	(5) Tomas, Do, Blondell, de Vries, Nguyen	TB A lack of symptoms despite contact with infected persons can lead migrants to place less value on prevention and screening (Tomas) HBV/HCV Apparent good health and personal preferences of migrants may discourage screening and vaccination (Do) HIV African and Latin migrants reported waiting until health crises, symptoms, or being extremely sick before seeking formal healthcare (Blondell) Feeling healthy and a lack of symptoms were consistently cited as barriers to HIV screening (Blondell)

**Table 3:** GRADE CERQual Evidence Profile.

Key Finding	Studies Supporting Key Finding	Methodological Quality	Relevance-Research Question	Relevance-Population	Coherence	Adequacy-Reviews	Adequacy-Primary Studies	Overall Assessment of Confidence	Explanation of Judgement
Subjects may be reluctant to undergo screening due to negative indirect costs of having a positive result—on employment status, immigration status, and social status	[20,21,24,26,29,32]	Moderate methodological concerns	No relevance concernsFull (6/6)	Moderate relevance concerns Full (3/6) partial (3/6)	Minor coherence concerns Coherent (5/6) Among Latino migrants in Spain, legal and administrative fears were not found to be significant barriers [29]	Minor adequacy concerns 6 reviews	20 studies	Low confidence	Lack of adequate evidence, including contradictory evidence, in addition to methodological concerns among reviews reporting this finding.
Patients value testing and treatment less if they are asymptomatic	[24,26,29,30,31]	Moderate methodological concerns	Minor relevance concerns Full (4/5) Indirect (1/5)	Moderate relevance concerns Full (2/5) Partial (3/5)	No coherence concerns Coherent (5/5)	Minor adequacy concerns 5 reviews	25 studies	Low confidence	Methodological concerns, indirect/partial relevance of reviews supporting key finding.
Incorrect knowledge of infectious diseases and low self-perceived risk are barriers to acceptability of screening and vaccination	[20,21,24,25,26,27,28,29,30,31,32]	Moderate methodological concerns	Minor relevance concerns Full (8/11) Indirect (3/11)	Moderate relevance concerns Full (8/11) Partial (3/11)	Minor coherence concerns Coherent (10/11) Perceiving tuberculosis as a severe disease (OR 0.29, 95% CI 0.09-0.91) was associated with refusal of TST screening [28]	Minor adequacy concerns11 reviews	81 studies	Moderate confidence	Some reviews have significant methodological concerns, yet the key finding is consistently supported by directly relevant data in reviews with only minor methodological concerns.
The acceptability of screening and treatment interventions is highly dependent on the cultural sensitivity and relationship with healthcare professionals	[20,21,24,25,26,27,28,29,30,31,32]	Moderate methodological concerns	Minor relevance concerns Full (10/11) Indirect (1/11)	Minor relevance concerns Full (8/11) Partial (3/11)	No coherence concerns Coherent (11/11)	Minor adequacy concerns 11 reviews	67 studies	Moderate confidence	Supported by all reviews. Although some reviews have significant methodological concerns, reviews with few methodological concerns report directly relevant data.
Stigma associated with infectious diseases is a barrier to the acceptability of screening interventions	[20,21,24,25,26,27,29]	Moderate methodological concerns	No relevance concerns Full (7/7)	Minor relevance concerns Full (6/7) Partial (1/7)	Minor coherence concerns Coherent (6/7) Stigma is not a significant factor in all studies. Two quantitative studies on stigma found it was not a significant deterrent to testing	Minor adequacy concerns 7 reviews	71 studies	Moderate confidence	Well-supported by review data that is directly relevant. Direct support from reviews with few methodological concerns.

Objective: To identify, appraise and synthesize review level evidence on values and preferences for infectious disease interventions among migrants in Europe. Perspectives: Experience and attitudes of migrant population regarding ID interventions in the EU/EEA? Included programs: Reviews of programs of testing and prevention of infectious diseases in migrants where values and preferences are evaluated.

**Table 4:** Summary CERQual Confidence Ratings.

Key Finding	CERQual Assessment Rating for Assessment of Confidence	Explanation of Confidence Rating
Incorrect knowledge of infectious diseases and low self-perceived risk are barriers to acceptability of screening and vaccination	Moderate confidence	Some reviews have significant methodological concerns, yet the key finding is consistently supported by directly relevant data in reviews with only minor methodological concerns.
The acceptability of screening and treatment interventions is highly dependent on the cultural sensitivity and sense of trust in healthcare professionals and their recommendations	Moderate confidence	Supported by all reviews. Although some reviews have significant methodological concerns, reviews with few methodological concerns report directly relevant data.
Stigma associated with infectious diseases is a barrier to the acceptability of screening interventions	Moderate confidence	Well-supported by review data that is directly relevant. Direct support from reviews with only mild methodological concerns.
Subjects may be reluctant to undergo screening due to negative indirect costs of having a positive result—on employment status, immigration status, and social status	Low confidence	Lack of adequate evidence, including contradictory evidence, in addition to methodological concerns among reviews reporting this finding.
Patients value testing and treatment less if they are asymptomatic	Low confidence	Methodological concerns, indirect/partial relevance of reviews supporting key finding.

## Supplementary Materials

The following are available online at http://www.mdpi.com/1660-4601/15/11/2329/s1, Table S1: Search Strategy.

## Appendix A. Determinants of Interest

We analyzed data on three overarching determinants of intervention uptake—values of main outcomes, acceptability, and accessibility. These are defined below:

Values of Main Outcomes of Infectious Disease Interventions:

- The importance placed upon the main outcomes of an intervention. These outcomes include those directly related to the disease (e.g., cure, symptom reduction, diagnosis), or costs or benefits resulting from the downstream effects of the intervention (e.g., side effects, time spent at the hospital, stigma, disclosure of disease status, cultural beliefs)

Acceptability of Infectious Disease Interventions:

- The willingness of the patient to request or adhere to the intervention based on their subjective attitudes and preferences towards the intervention itself or the process of receiving it (e.g., adherence challenges, social/cultural attitudes, fears about the procedure)

Accessibility of Infectious Disease Interventions:

- The ease with which patients use an infectious disease intervention. Determinants of accessibility include policies, community factors, healthcare service organization, or the delivery of the intervention itself.

## Appendix B. Determinants of Interest

- Study design: Systematic reviews (qualitative or qualitative/quantitative) defined as any review that includes selection criteria, search strategy, and use of at least one database
- Time: Published after 1 January 2010
- Language: English language
- Relevant to the PICO question:
  - Population: Migrants from Low- and Middle-Income Countries residing in High-Income Countries (i.e., permanent resettlement countries)
  - Intervention: Prevention, screening, and treatment interventions for infectious diseases (tuberculosis, hepatitis, VPDs, HIV, parasitic diseases)
  - Comparison: No intervention
  - Outcome: Valuation of outcomes, views about acceptability and accessibility of interventions

## Appendix C. Data Abstraction Tables

**Table A1:** Value of Outcomes.

Citation
Disease
Knowledge of Disease Status
Behavioral Prevention
Vaccination
Treatment of Asymptomatic Disease
Cure of Symptomatic Disease

**Table A2:** Acceptability.

Citation
Demand-Side Determinants
User’s attitudes and Expectations
Household attitudes and expectations
Information on healthcare choice/providers
Disease-related knowledge
Intervention-related knowledge
Stigma
Indirect costs
Acculturation
SocialSupply-Side Determinants
Characteristics of the Health Services
Management/Staff Efficiency
Technology
Staff Interpersonal Skills, Including Trust
Wages and Quality of Staff
Language Barriers

**Table A3:** Accessibility.

Citations
Demand-Side Determinants
Indirect costs to household (e.g. transport, legal status)
Household income and willingness to pay
Opportunity costs
Means of transport available
System navigation
Low self-esteem and little assertivenessSupply-Side Determinants
Service/household location
Availablity of health workers, drugs, equipment
Direct price of service, including informal fees
Waiting time
Unqualified health woerks, absenteeism
Non-integration of health services
Lack of opportunity (exclusion from services)
Late or no referral
