# Supplementary material for: Accessibility and Acceptability of Infectious Disease Interventions Among Migrants in the EU/EEA: A CERQual Systematic Review
Source: Int J Environ Res Public Health. 2018 Oct 23;15(11):2329. doi: 10.3390/ijerph15112329 (PMC6267477; doi:10.3390/ijerph15112329)
Supplement: Supplementary file 1 [file ijerph-15-02329-s001.pdf]

Database: Ovid MEDLINE(R) 1946 to Present with Daily Update
Search Date: 29 July 2016

----

1 exp "Emigrants and Immigrants"/ (7985)

2 (emigra$ or immigra$ or migrant$ or migration$ or newcomer$ or refugee$).mp. (228210)

3 (asylum adj2 (seek$ or sought)).tw. (969)

4 (displaced adj2 (people or person?)).tw. (570)

5 or/1-4 (228418)

6 Health Services Accessibility/ (58905)

7 Health Equity/ (98)

8 ((availab$ or access$ or barrier$ or equit$) adj3 (care or health$)).mp. (90405)

9 "Patient Acceptance of Health Care"/ (35583)

10 exp Patient Satisfaction/ (70835)

11 ((consumer? or patient?) adj3 (accept$ or attitud$ or satisf$) adj3 (care or health or healthcare)).mp. (39311)

12 or/6-11 (188999)

13 exp Communicable Diseases/ (30347)

14 (infectious adj2 disease?).mp. (86383)

15 ((communicable or contagious or transmissible) adj2 (disease? or infection?)).mp. (54011)

16 or/13-15 (128068)

17 exp Hepatitis B/ (50672)

18 (CHB or HBV or HepB).mp. (32209)

19 ((hep or hepatitis) adj3 B).mp. (82837)

20 hbsag.tw. (16167)

21 (hbs adj2 ag).tw. (721)

22 hb-s-ag.tw. (24)

23 ((serum or type b) adj2 hepatitis).mp. (3435)

24 or/17-23 [Hep B] (86842)

25 exp Hepatitis C/ (53765)

26 (CHC or HCV or HepC).mp. (44016)

27 ((hep or hepatitis) adj3 C).mp. (71676)

28 or/25-27 [Hep C] (75877)

29 (hiv or hiv1$ or hiv2$).mp. (284241)

30 (human adj (immunedeficienc$ or immune deficienc$ or immunodeficienc$ or immuno deficienc$)).mp. (82014)

31 Acquired Immunodeficiency Syndrome/ (74557)

32 (acquired adj (immunedeficienc$ or immune deficienc$ or immunodeficienc$ or immuno deficienc$)).mp. (88461)

33 aids.hw. (60564)

34 (aids adj2 (infect$ or virus$)).mp. (6042)

35 or/29-34 [hiv] (356227)

36 exp Schistosoma/ (15807)

37 bilharzia$.tw. (2438)

38 exp Schistosomiasis/ (21713)

39 schistosom$.mp. (30488)

40 katayama fever$.tw. (30)

41 Strongyloides/ (995)

42 Strongyloides stercoralis/ (1072)

43 Strongyloidiasis/ (3337)

44 strongyloid$.mp. (5559)

45 or/36-44 [IP] (35821)

46 Tuberculosis/ (97611)

47 tuberculo$.mp. (220149)

48 tb.tw. (29844)

49 ltbi.tw. (1123)

50 or/46-49 [TB] (226741)

51 exp Measles/ (14334)

52 exp Measles virus/ (6143)

53 measles.mp. (23714)

54 (rubeola or rubeolla).tw. (305)

55 Mumps/ (4253)

56 Mumps virus/ (1974)

57 mumps.mp. (9610)

58 ((epidemic or infectious) adj parotiti$).tw. (395)

59 exp Rubella/ (7779)

60 Rubella virus/ (3332)

61 rubella?.mp. (13919)

62 german measles.tw. (204)

63 mmr.mp. (5350)

64 exp Poliomyelitis/ (19326)

65 Poliovirus/ (9420)

66 polio$.mp. (32504)

67 Tetanus/ (9191)

68 tetanus.mp. (25655)

69 Diphtheria/ (6558)

70 Diphtheria toxin/ (3381)

71 diphtheria?.mp. (18767)

72 (dtp or dtap or tdap).tw. (2063)

73 Whooping Cough/ (7628)

74 whooping cough.mp. (8271)

75 Bordetella pertussis/ (4818)

76 pertuss$.mp. (27187)

77 Meningitis, Haemophilus/ (2410)

78 Haemophilus influenzae/ (12024)

79 Haemophilus influenzae type b/ (1061)

80 ((haemophilus or hemophilus) adj influenzae).mp. (19828)

81 "h. influenzae".tw. (5962)

82 hib.tw. (2616)

83 or/51-82 [Vacc] (148344)

84 12 or 16 or 24 or 28 or 35 or 45 or 50 or 83 (1130618)

85 meta analysis.mp,pt. (99896)

86 review.pt. (2085646)

87 search$.tw. (268070)

88 or/85-87 (2278940)

89 5 and 84 and 88 (1972)

90 animals/ not (humans/ and animals/) (4249861)

91 89 not 90 (1921)

92 (2010$ or 2011$ or 2012$ or 2013$ or 2014$ or 2015$ or 2016$).em. (5392589)

93 91 and 92 (769)

94 remove duplicates from 93 (741)

****

Database: Embase <1974 to 2016 July 28>
Search Date: 29 July 2016

----

1 exp migrant/ (23718)

2 (emigra$ or immigra$ or migrant$ or migration$ or newcomer$ or refugee$).mp. (348985)

3 (asylum adj2 (seek$ or sought)).tw. (1254)

4 (displaced adj2 (people or person?)).tw. (686)

5 or/1-4 (349482)

6 health care access/ (44591)

7 ((availab$ or access$ or barrier$ or equit$) adj3 (care or health$)).mp. (104999)

8 exp patient attitude/ (302067)

9 ((consumer? or patient?) adj3 (accept$ or attitud$ or satisf$) adj3 (care or health or healthcare)).mp. (5925)

10 or/6-9 (399274)

11 communicable disease/ (19379)

12 (infectious adj2 disease?).mp. (94520)

13 ((communicable or contagious or transmissible) adj2 (disease? or infection?)).mp. (33572)

14 or/11-13 (121783)

15 exp hepatitis B/ (80896)

16 (CHB or HBV or HepB).tw. (54027)

17 ((hep or hepatitis) adj3 B).tw. (92522)

18 hbsag.tw. (24478)

19 (hbs adj2 ag).tw. (1141)

20 hb-s-ag.tw. (626)

21 ((serum or type b) adj2 hepatitis).tw. (4229)

22 or/15-21 [Hep B] (129168)

23 exp hepatitis C/ (88369)

24 (CHC or HCV or HepC).tw. (76347)

25 ((hep or hepatitis) adj3 C).tw. (93306)

26 or/23-25 [Hep C] (131347)

27 (hiv or hiv1$ or hiv2$).mp. (320981)

28 exp human immunodeficiency virus infection/ (326876)

29 exp human immunodeficiency virus/ (159417)

30 (human adj (immunedeficienc$ or immune deficienc$ or immunodeficienc$ or immuno deficienc$)).tw. (82582)

31 (acquired adj (immunedeficienc$ or immune deficienc$ or immunodeficienc$ or immuno deficienc$)).tw. (22410)

32 aids.hw. (11242)

33 (aids adj2 (infect$ or virus$)).tw. (6854)

34 or/27-33 [hiv] (455619)

35 exp schistosoma/ (21829)

36 bilharzia$.tw. (2496)

37 exp schistosomiasis/ (22106)

38 schistosom$.tw. (29237)

39 katayama fever$.tw. (42)

40 strongyloides/ (1248)

41 strongyloides stercoralis/ (2473)

42 strongyloidiasis/ (4019)

43 strongyloid$.tw. (5024)

44 or/35-43 [IP] (42644)

45 tuberculosis/ (116934)

46 tuberculo$.tw. (208521)

47 tb.tw. (46163)

48 ltbi.tw. (1971)

49 or/45–48 [TB] (253812)

50 measles/ (18067)

51 measles virus/ (8427)

52 measles.tw. (22452)

53 (rubeola or rubeolla).tw. (377)

54 mumps/ (7075)

55 mumps virus/ (2392)

56 mumps.tw. (8159)

57 ((epidemic or infectious) adj parotiti$).tw. (395)

58 rubella/ (10692)

59 rubella virus/ (4553)

60 rubella?.tw. (12936)

61 german measles.tw. (263)

62 mmr.tw. (9055)

63 poliomyelitis/ (21756)

64 exp poliomyelitis virus/ (9786)

65 polio$.mp. (39999)

66 tetanus/ (14535)

67 tetanus.tw. (23217)

68 diphtheria/ (10792)

69 diphtheria toxin/ (4666)

70 diphtheria?.tw. (15223)

71 (dtp or dtap or tdap).tw. (2887)

72 pertussis/ (12247)

73 pertuss$.tw. (27104)

74 whooping cough.tw. (3269)

75 haemophilus meningitis/ (385)

76 exp haemophilus influenzae/ (26827)

77 ((haemophilus or hemophilus) adj influenzae).tw. (19221)

78 "h. influenzae".tw. (7325)

79 hib.tw. (3362)

80 or/50-79 [Vacc] (180188)

81 10 or 14 or 22 or 26 or 34 or 44 or 49 or 80 (1532838)

82 meta analys$.mp. (176722)

83 search$.tw. (381239)

84 review.pt. (2187317)

85 or/82-84 (2546877)

86 5 and 81 and 85 (3301)

87 (exp animal/ or animal.hw. or nonhuman/) not (exp human/ or human cell/ or (human or humans).ti.) (5909424)

88 86 not 87 (3149)

89 (2010$ or 2011$ or 2012$ or 2013$ or 2014$ or 2015$ or 2016$).dd. (9919671)

90 88 and 89 (1426)

91 remove duplicates from 90 (1392)

****

Databases: Database of Abstracts of Reviews of Effects (DARE) and Cochrane Database of Systematic Reviews (CDSR) and NHS EED
Search Date: 29 July 2016

ID Search
#1 MeSH descriptor: [Emigrants and Immigrants] explode all trees
#2 MeSH descriptor: [Transients and Migrants] explode all trees
#3 (emigra* or immigra* or migrant* or migration* or newcomer* or refugee*)
#4 asylum near/2 (seek* or sought)
#5 displaced near/2 (people or person*)
#6 {or #1-#5}
#7 MeSH descriptor: [Health Services Accessibility] this term only
#8 MeSH descriptor: [Health Equity] this term only
#9 (availab* or access* or barrier* or equit*) near/3 (care or health*)
#10 MeSH descriptor: [Patient Acceptance of Health Care] this term only
#11 MeSH descriptor: [Patient Satisfaction] explode all trees
#12 (consumer* or patient*) adj3 (accept* or attitud* or satisf*) near/3 (care or health or healthcare)
#13 {or #7-#12}
#14 MeSH descriptor: [Communicable Diseases] explode all trees
#15 infectious near/2 disease*
#16 (communicable or contagious or transmissible) near/2 (disease* or infection*)
#17 {or #14-#16}
#18 MeSH descriptor: [Hepatitis B] explode all trees
#19 (CHB or HBV or HepB)
#20 (hep or hepatitis) near/3 B
#21 hbsag:ti,ab
#22 hbs near/2 ag:ti,ab
#23 hb-s-ag:ti,ab
#24 (serum or type b) near/2 hepatitis:ti,ab
#25 {or #18-#24}
#26 MeSH descriptor: [Hepatitis C] explode all trees
#27 (CHC or HCV or HepC)
#28 (hep or hepatitis) near/3 C
#29 {or #26-#28}
#30 (hiv or hiv1* or hiv2*)
#31 human next (immunedeficienc* or immune deficienc* or immunodeficienc* or immuno deficienc*):ti,ab
#32 MeSH descriptor: [Acquired Immunodeficiency Syndrome] this term only
#33 acquired next (immunedeficienc* or immune deficienc* or immunodeficienc* or immuno deficienc*):ti,ab
#34 aids:kw
#35 aids near/2 (infect* or virus*):ti,ab
#36 {or #30-#35}
#37 MeSH descriptor: [Schistosoma] explode all trees

#38 bilharzia*
#39 MeSH descriptor: [Schistosomiasis] explode all trees
#40 schistosom*
#41 katayama fever
#42 MeSH descriptor: [Strongyloides] this term only
#43 MeSH descriptor: [Strongyloides stercoralis] this term only
#44 MeSH descriptor: [Strongyloides] this term only
#45 strongyloid*
#46 {or #37-#45}
#47 MeSH descriptor: [Tuberculosis] this term only
#48 tuberculo*
#49 tb:ti,ab
#50 ltbi:ti,ab
#51 {or #47-#50}
#52 (measles or rubeola or rubeolla)
#53 (mumps or epidemic parotiti* or infectious parotiti*)
#54 (rubella* or german measles)
#55 mmr
#56 polio*
#57 tetanus
#58 (diphtheria* or dtp or dtap or tdap)
#59 (pertuss* or whooping cough)
#60 (haemophilus meningitis or hemophilus meningitis)
#61 (haemophilus influenzae or (hemophilus influenzae))
#62 h. influenzae
#63 hib
#64 {or #52-#63}
#65 #13 or #17 or #25 or #29 or #36 or #46 or #51 or #64
#66 #6 and #65
#67 #66 in Cochrane Reviews (Reviews and Protocols)
#68 #66 in Other Reviews

****

Database: EBSCO CINAHL <1970 to July 2016>
Search Date: 29 July 2016

#	Query	Limiters/Expanders	Last Run Via	Results
S70	S66 AND S69	Limiters - Exclude MEDLINE records		77
S69	S67 OR S68			1,407,771
S68	EM 2010 or EM 2011 or EM 2012 or EM 2013 or EM 2014 or EM 2015 or EM 2016			1,313,069
S67	PY 2010 or PY 2011 or PY 2012 or PY 2013 or PY 2014 or PY 2015 or PY 2016			1,312,169
S66	(S5 AND S60 AND S65)			522
S65	S61 OR S62 OR S63 OR S64			58,668
S64	(TI meta analy* or AB meta analy*)			18,461
S63	(MH "Meta Analysis")			17,583
S62	PT systematic review			38,804

S61 (MH "Systematic Review") 26,076
S60 (S10 OR S14 OR S22 OR S26 OR S34 OR S41 OR S46 OR S59) 351,140
S59 (S47 OR S48 OR S49 OR S50 OR S51 OR S52 OR S53 OR S54 OR S55 OR S56 OR S57 OR S58) 21,664
S58 TX hib 1,394
S57 TX "h. influenzae" 1,320
S56 TX (haemophilus influenzae OR haemophilus meningitis OR hemophilus influenzae OR hemophilus meningitis) 2,614
S55 TX (pertuss* OR whooping cough) 5,258
S54 TX (diphtheria OR dtp OR dtap OR tdap) 4,575
S53 TX tetanus 5,620
S52 TX polio* 7,967
S51 (MH "Poliomyelitis+") 2,137
S50 TX (rubella* OR german measles) 5,292
S49 TX (epidemic parotiti* OR infectious parotiti*) 16
S48 TX mumps 4,492
S47 TX (measles OR rubeola OR rubeolla) 7,659
S46 (S42 OR S43 OR S44 OR S45) 35,833
S45 TX ltbi 315
S44 TX tb 20,924
S43 TX tuberculo* 22,046
S42 (MH "Tuberculosis") 6,869
S41 (S35 OR S36 OR S37 OR S38 OR S39 OR S40) 2,852
S40 TX strongyloid* 233
S39 (MH "Helminthiasis+") 2,152
S38 TX katayama fever 9
S37 TX bilharzia* 82
S36 TX schistosom* 1,074
S35 (MH "Schistosomiasis+") 418
S34 S27 OR S28 OR S29 OR S30 OR S31 OR S32 OR S33 108,053
S33 TX aids N2 (infect* or virus*) 8,087
S32 MW aids 18,755
S31 TX acquired N1 (immunedeficienc* or immune deficienc* or immunodeficienc* or immuno deficienc*) 17,616
S30 (MH "Acquired Immunodeficiency Syndrome") 11,846
S29 TX human N1 (immunedeficienc* or immune deficienc* or immunodeficienc* or immuno deficienc*) 17,458
S28 TX (hiv or hiv1* or hiv2*) 92,725
S27 (MH "Human Immunodeficiency Virus+") 4,228
S26 S23 OR S24 OR S25 16,479
S25 TX (hep or hepatitis) N3 C 14,430
S24 TX (CHC or HCV or HepC) 6,359
S23 (MH "Hepatitis C+") 6,858
S22 (S15 OR S16 OR S17 OR S18 OR S19 OR S20 OR S21) 14,773
S21 TX (serum or type b) N2 hepatitis 1,169
S20 TX hb-s-ag 2
S19 TX hbs N2 ag 31

S18 TX hbsag 931
S17 TX (hep or hepatitis) N3 B 13,134
S16 TX (CHB or HBV or HepB) 4,353
S15 (MH "Hepatitis B+") 4,314
S14 S11 OR S12 OR S13 63,766
S13 TX (communicable or contagious or transmissible) N2 (disease* or infection*) 14,327
S12 TX (infectious N2 disease*) 54,715
S11 (MH "Communicable Diseases") 5,897
S10 (S6 OR S7 OR S8 OR S9) 176,323
S9 TX (consumer* or patient*) N3 (accept* or attitud* or satisf*) N3 (care or health or healthcare) 12,735
S8 (MH "Patient Satisfaction") 27,662
S7 TX ((availab* or access* or barrier* or equit*) N3 (care or health*) 145,137
S6 (MH "Health Services Accessibility+") 46,693
S5 S1 OR S2 OR S3 OR S4 56,420
S4 TX (asylum N2 seek*) OR TX (asylum N2 sought) 1,884
S3 TX (emigra* or immigra* or migrant* or migration* or newcomer* or refugee*) 56,050
S2 (MH "Refugees") 3,603
S1 (MH "Transients and Migrants") or (MH Immigrants) 9,514

****
